# Rapid Biotic and Abiotic Transformation of Toxins produced by *Ostreopsis.* cf. *ovata*

**DOI:** 10.3390/md20120748

**Published:** 2022-11-28

**Authors:** Eva Ternon, Olivier P. Thomas, Rodolphe Lemée, William H. Gerwick

**Affiliations:** 1 Center for Marine Biotechnology and Biomedicine, Scripps Institution of Oceanography, University of California, La Jolla, CA 92037, USA; 2 Laboratoire d’Océanographie de Villefranche (UMR 7093), Sorbonne Université, CNRS, 06230 Villefranche-Sur-Mer, France; 3 School of Biological and Chemical Sciences, Ryan Institute, University of Galway, University Road, H91TK33 Galway, Ireland; 4 Skaggs School of Pharmacy and Pharmaceutical Sciences, University of California, La Jolla, CA 92037, USA

**Keywords:** ova- and liguriatoxins, catabolites, metabolomics, *Ostreopsis* cf. *ovata*

## Abstract

The dinoflagellate *Ostreopsis* cf. *ovata* produces several families of toxic polyketides. Despite only a few field measurements of these phycotoxins in seawater and aerosols, they are believed to be responsible for dermatitis and the toxic inhalations reported during blooms of this species. Therefore, the stability of these compounds in seawater is essential to understanding the causes of these symptoms, however, this has never been assessed. In the current study, the optimization of a solid phase extraction (SPE) procedure was first performed to ensure the most efficient extraction of all phycotoxins known to be produced by this strain, including the recently described liguriatoxins. The SPE cartridge SDBL^®^ under non acidified conditions offered the best option. The stability of the ovatoxins and the liguriatoxins under biotic and abiotic stress was assessed by exposing the spent medium of a culture of *Ostreopsis* cf. *ovata* to its bacterial consortium and natural sunlight. A rapid biotic transformation was detected for both families of compounds. When exposed to bacteria, the half-lives of the ovatoxins were reached before 10 h and at 36 h, 97% of these toxins had been transformed. The half-lives of the liguriatoxins were 10 h under these conditions. Photolysis (abiotic degradation) of the ovatoxins (T_1/2_ < 36 h) was faster than for the liguriatoxins (T_1/2_ > 62 h). Although none of the catabolites of these phycotoxins were thoroughly identified, an untargeted metabolomics approach combined with molecular networking highlighted the presence of several compounds exhibiting structural similarities with the ovatoxins. Additional work should confirm the preliminary findings on these potential ovatoxins’ catabolites and their biological properties. The rapid transformation of *O*. cf. *ovata*’s phycotoxins introduces questions concerning their presence in seawater and their dispersion in the sea spray aerosols. The compounds involved in the toxic inhalations and dermatitis often experienced by beachgoers may stem from the catabolites of these toxins or even unrelated and as yet unidentified compounds.

## 1. Introduction

The toxic dinoflagellate *Ostreopsis* cf. *ovata* is regularly associated with toxic outbreaks causing dermatitis and respiratory syndromes in populations living close to the seashore [1,2,3,4]. The toxicity of these blooms is generally attributed to the production of various palytoxin (PLTX) analogs named ovatoxins (OVTX) [1,5,6,7], but could also result from the newly described liguriatoxins (LGTX) and rivieratoxins (RVTX) [8]. These two types of toxicity symptoms that involve either direct or indirect contact with seawater suggest that the toxins are waterborne. Indeed, sea spray aerosols form from breaking waves, bursting bubbles and wind interaction with the ocean surface. Therefore, their organic content is mainly determined by the molecular composition of the upper water column [9,10,11]. In controlled systems, a natural and continuous extracellular release of OVTXs occurs throughout the growth of *O*. cf. *ovata* [12,13,14], and these waterborne OVTXs were shown to be transferred to sea spray aerosols at specific growth stages of the microalgae in controlled systems [15]. Yet, field detection of OVTX in the water column and in aerosols has been rare, even during events of high cell proliferation and toxin production [16,17]. This observation raises the question concerning the fate of the OVTXs once they are released in seawater and submitted to in situ abiotic and biotic factors.

Microalgal exometabolomes are rich in diverse compounds [18,19,20] and strongly interact with their associated bacterial consortium [21,22,23]. Most studies have focused on the uptake of small soluble compounds easily assimilated by bacteria, such as amino-acids, sugars, glucose, osmolytes, vanillic acid and dimethylsulfidepropionate [24,25,26,27,28]. While phycotoxins are also a constituent of microalgal exometabolomes that could be taken up by bacteria and biotransformed [29,30,31], this process has been generally overlooked.

In addition, dissolved organic compounds in the ocean surface, like phycotoxins, can be transformed through abiotic processes such as solar irradiation. In particular, the colored fraction of dissolved organic matter (cDOM) absorbs light in the UV and visible regions and participates in a multitude of photochemical reactions [32]. Photolysis yields organic molecules with modified structures and properties [33,34,35], such as low molecular weight organic acids [36] and other reactive intermediates [37,38]. Aromatics are particularly photolabile and are more easily degraded relative to non-aromatic ones [39]. Still, non-aromatic molecules, including several phycotoxins, are also prone to photolysis [40,41,42,43]. Due to the investigation into breakdown products being relatively new, these compounds have not been considered when investigating the toxicity of the relevant harmful algal blooms.

*Ostreopsis* cf. *ovata*’s toxins are large molecular weight polyketides containing multiple hydroxy groups of a non-aromatic nature, although OVTX contain chromophoric moieties [5,6,8]. *Ostreopsis* cf. *ovata* is a benthic species that inhabits shallow waters and often develops on the surface of macroalgae [44], but can be present as a planktonic phase [45,46]. During high proliferation events, these toxic cells abundantly occupy the first two meters of the water column [44,47] where the toxins may be released and are therefore submitted to high solar radiation and bacterial uptake. The concentration of waterborne toxins in the water column has been assessed a few times using a liquid–liquid extraction protocol with butanol [16] that requires high volumes of solvent, especially when processing large volumes of seawater. The use of solid phase extraction (SPE) is another method by which to extract and concentrate organic compounds from seawater [48] and involves a first step of sorbent selection to optimally extract the targeted compounds. To date, two sorbents, StrataX and BondElut C_18_, only provided low percentage recoveries for OVTX-a [49]; therefore, the efficiency of other sorbents needs to be evaluated for their efficiency in extracting *O*. cf. *ovata*’s toxins.

Here, we seek to better understand the transformation of the different *O*. cf. *ovata* toxins through bacterial activity (biotic transformation) and photolysis (abiotic transformation). Studying the sensitivity of the *O*. cf. *ovata*’s toxins to biotic and abiotic transformation is essential to better understand their diffusion in seawater and subsequent transfer to sea spray aerosols, and ultimately the toxicity of *Ostreopsis* blooms towards coastal human populations. As a first step, the optimization of an extraction protocol for the waterborne toxins was undertaken. The transformation of these toxins was investigated by assessing the evolution of their concentration under biotic and abiotic pressure. Their by-products were detected using an un-targeted metabolomics approach and molecular networking in order to propose biomarkers of these transformation pathways.

## 2. Results

### 2.1. Cartridge Selection and Toxin Composition of O. cf. ovata’s Spent Medium

**Figure 1:**
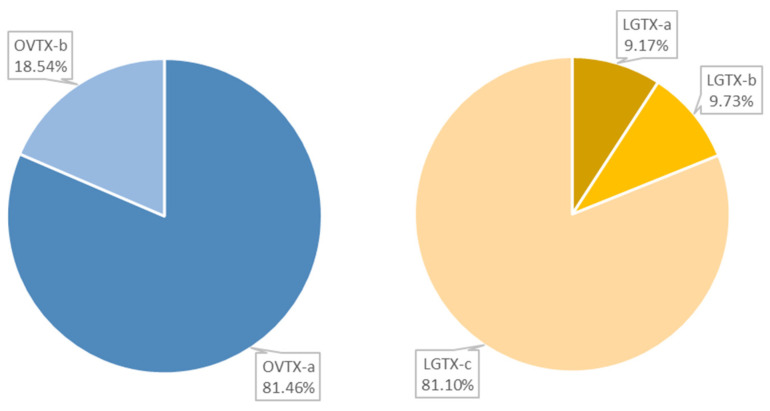
Relative waterborne OVTX and LGTX compositions in 10-day old spent culture medium of *O.* cf. *ovata* assessed by Ultra-High Pressure Liquid Chromatography coupled to High Resolution Mass Spectrometry (UHPLC-HRMS).

The different toxin families known to be produced by *O*. cf. *ovata* strains, including the ovatoxins (OVTX), the mascarenotoxins (MCTX), the ostreols (OSTR) as well as the newly described liguriatoxins (LGTX) and the rivieratoxins (RVTX), were screened for in the spent medium. Using their MS and MS^2^ fragmentation patterns, the following toxins were identified: RVTX-c ([M + H + Na]^2+^ ion at *m*/*z* 697.4740), the OVTX-a -and -b, ([M + 2H − H_2_O]^2+^ ions at *m*/*z* 1315.2489 and 1337.2633, respectively) as well as the LGTX-a, -b and –c ([M + 2H]^2+^ ions at *m*/*z* 987.0349, 1043.0670, 1072.0673, respectively). The total OVTX content was comprised of 81.46% of OVTX-a and 18.54% of OVTX-b (Figure 1). The LGTX-c contributed to the largest share of the LGTX content, with 81.10%, against 9.17% and 9.73%, for LGTX-a and -b, respectively (Figure 1). Very low intensities of the RVTX were detected, therefore, only OVTX and LGTX were considered in subsequent experiments.

**Figure 2:**
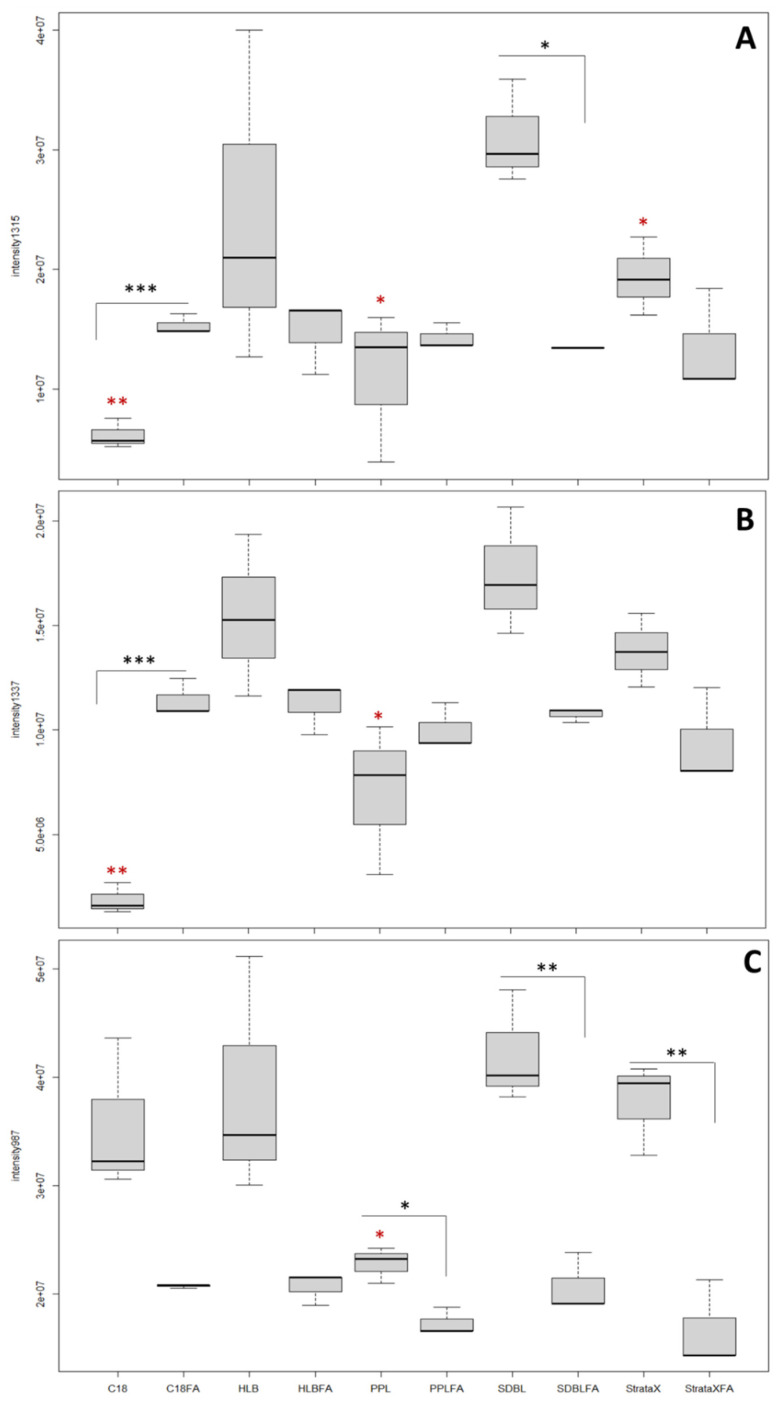

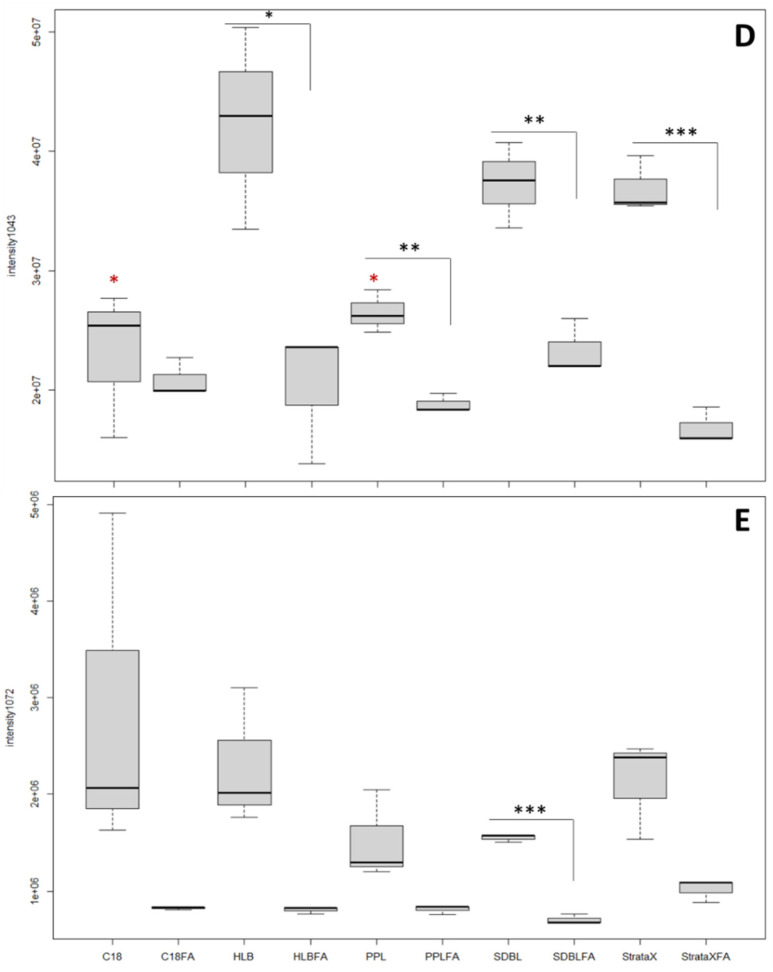
Boxplots comparing the mean intensities of the ions (**A**) *m*/*z* 1315.2546 (OVTX-a), (**B**) *m*/*z* 1337.2705 (OVTX-b), (**C**) *m*/*z* 987.0342 (LGTX-a), (**D**) *m*/*z* 1043.0673 (LGTX-b) and (**E**) *m*/*z* 1072.0619 (LGTX-c), in acidified (FA) and non-acidified samples. Sorbents are C18 (octadecyl bonded silica), HLB (Polymeric Phase), PPL (Styrene Divinyl Benzene), SDBL (Styrene Divinyl Benzene), StrataX (Polymeric phase). Statistical significance was assessed with the following threshold: * 0.05 < *p* < 0.5; ** 0.005 < *p* < 0.05, *** *p* < 0.005. Black stars compare acidified versus non acidified conditions for one sorbent type, whereas red stars compare one sorbent type to the SDBL sorbent.

At unmodified pH, most of the toxins exhibited a higher affinity for the sorbents HLB, SDBL and StrataX (Figure 2). The SDBL cartridge achieved a significantly higher extraction efficiency compared with the PPL and the C18 sorbents for the OVTX-a, -b and the LGTX-a, -b (0.005 < *p* < 0.05). No significant differences extracting the LGTX-c were observed between the SDBL sorbents and the others. In HLB extracts, the toxin content displayed a high variability among replicates, making this sorbent not suitable for a precise quantification of solubilized toxins of *O*. cf. *ovata*, and in particular, OVTX-a. Conversely, a high reproducibility was achieved using the StrataX sorbents, although the extraction time was noticeably longer compared to other sorbents. In addition, the toxin content was systemically lower in StrataX extracts compared to SDBL extracts, although not statistically significant.

The addition of formic acid to the samples before their extraction did not improve the extraction efficiency of the dissolved toxins, regardless of the nature of the sorbent. By contrast, both the SDBL and StrataX sorbents displayed a significantly lower extraction efficiency for all OVTX and LGTX in acidic conditions (0.0002 < *p* < 0.05, Figure 2) while no significant differences were observed using the sorbents HLB and PPL for the two OVTXs. The extraction of the LGTX was decreased in the presence of acid when using both the PPL and HLB sorbents. The addition of acid modulated the extraction efficiency of the C18 sorbent in a compound specific fashion. An improved extraction of OVTX in the presence of acid was measured (*p* < 0.0002, Figure 2A), whereas this was not significant for the LGTX (Figure 2B,C). Therefore, by combining high extraction efficiency, limited processing extraction time and good replicability, the SDBL cartridge at unmodified pH was selected as the best sorbent for extraction of *O*. cf. *ovata*’s dissolved toxins in seawater samples.

### 2.2. Biotic and Abiotic Transformation

In the absence of authentic standards for quantification, a relative comparison of the chromatographic peak areas between treated and control conditions was performed to assess the biotic and abiotic transformation of the toxins.

The spontaneous transformation of *O.* cf. *ovata* toxins was controlled by incubating the waterborne toxins in sterile L1 medium in the dark (BLK) at room temperature over the course of the experiment (for 10, 36 and 62 h). Neither the OVTX nor the LGTX peak areas displayed significant differences (*p* > 0.05) in these BLK samples across the experiment and showed intensities values ranging from 0.3 to 1.7 × 10^7^ for OVTX, and from 10.4 to 16.6 × 10^7^ for LGTX (Figure S1).

**Figure 3:**
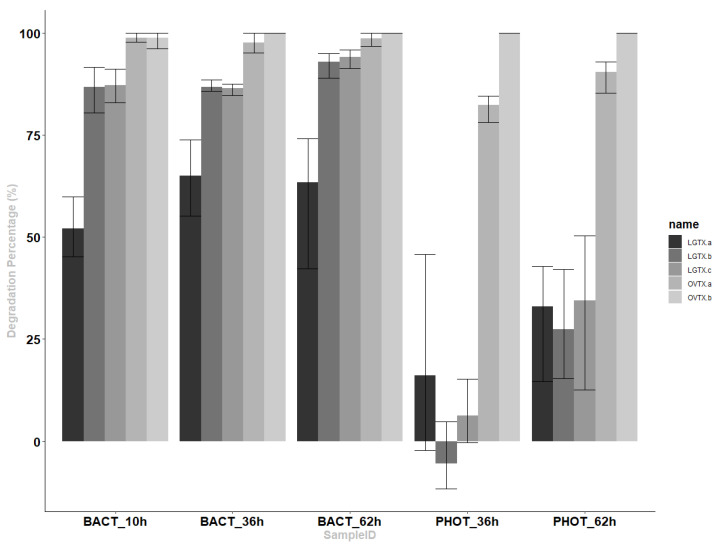
Degradation percentages normalized to control samples (BLK) collected at the same sampling times (%) and their standard deviation for LGTX-a, LGTX-b, LGTX-c, OVTX-a and OVTX-b, under conditions of bacteria exposure (BACT) at 10, 36 and 62 h or natural sunlight (PHOT) at 36 and 62 h.

Bacterial consortium (BACT) samples were incubated under artificial light, avoiding photolysis effects while maintaining normal light conditions for the bacteria. Nevertheless, we cannot exclude an effect of the artificial light on the toxins’ transformation. After only 10 h of incubation under a 14/10 h light cycle with the *O*. cf. *ovata*’s BACT, the peak areas of all toxins were considerably reduced compared to the blanks (*p* < 0.05, Figure 3). Meanwhile, the bacterial abundance increased throughout the experiment from 6.02 ± 0.24 × 10^6^ to 19.9 ± 2.5 × 10^6^ cell mL^−1^. Most of the transformation of the OVTX analogs occurred within the first 10 h, with up to 98.9 ± 1.1% and 98.74 ± 2.19% of OVTX-a and –b, transformed, respectively. After 24 h, the OVTX-b levels were under detection. A slower biodegradation of the LGTX was observed, and analogs displayed subtle differences. Only 52.14 ± 7.41% of LGTX-a had been transformed after 10 h of incubation compared with 86.81 ± 5.73 and 87.22 ± 4.12% for LGTX-b and –c, respectively. After 62 h, the transformation of all LGTX had increased, reaching 63.33 ± 18.20, 92.97 ± 3.45 and 94.08 ± 2.42% for LGTX-a, -b and -c, respectively.

Irradiation with natural sunlight (PHOT, Figure 3) induced a rapid transformation of OVTX-a and –b, reaching up to 82.38 ± 3.71% and 100% within 36 h (corresponding to two days of sunlight). At longer time points, OVTX-a continued to be transformed with 90.37 ± 4.36% degraded after 62 h. A significant transformation of the LGTXs by natural sunlight only occurred after 62 h and only reached 27.33 to 34.46% at that time point.

Therefore, the half-lives of the OVTX analogs are quite short under either bacterial consumption or solar irradiation (<10 and <36 h, respectively). LGTX’s half-lives were short under bacterial activity (~10 h) while they were not reached after three days of sunlight (62 h).

### 2.3. Biotoxin By-Products

The untargeted metabolomics analysis led to 402 features that could be segregated into two groups: (1) features lost, or (2) features gained after being exposed to either bacteria or sunlight. A list of the major ions contributing to these two groups was established (Table 1) using the variables of importance (VIP) obtained from a Partial Least Squares Discriminant Analysis (PLS-DA).

**Figure 4:**
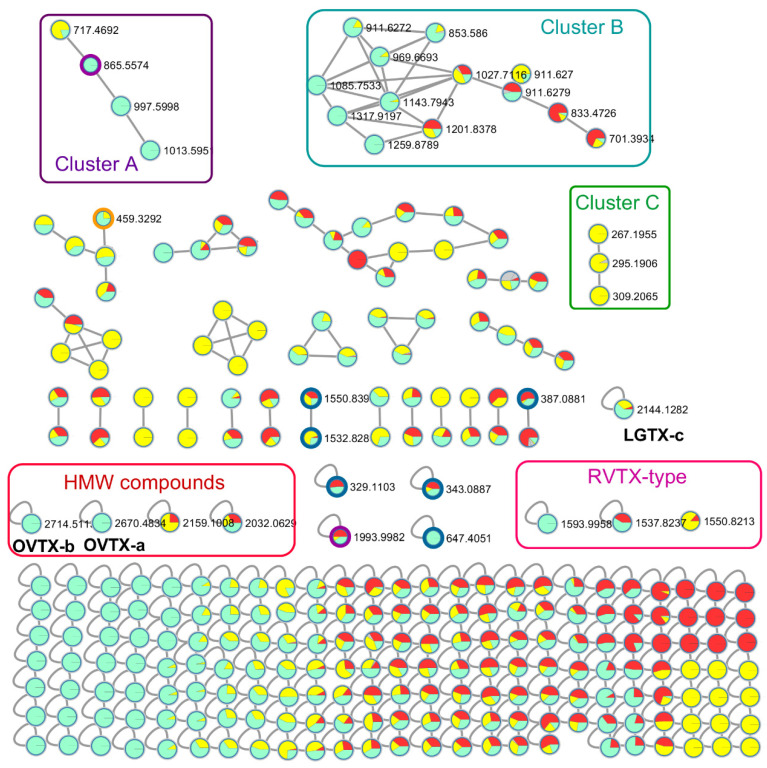
Molecular network representing the relationships between the ions detected in the three treatments: BLK (light blue), PHOT (yellow) and BACT (red). Grey pies represent either L1 media or analytical blanks. Rectangles mark the three clusters A, B and C mentioned in the text as well as two group of compounds, high molecular weight (>2000 Da, red) and RVTX-type ions (pink). The lost ions listed in Table 1 are marked with a colored border: dark blue for the PHOT treatment, orange for the BACT treatment and purple when the ions are lost in both PHOT and BACT treatments. The singletons corresponding to OVTX-a, OVTX-b and LGTX-c are also indicated.

Among the lost features, three ions were present in both the BACT and PHOT samples: feature 2 (*m*/*z* 647.4052), 6 (*m*/*z* 997.5004) and 8 (*m*/*z* 865.5573) (in BACT Lost columns, Table 1). Reasonably prominent MS^2^ fragments were obtained for features 2 and 6, allowing their inclusion in the molecular network, but it was not possible to firmly identify their structures. Despite a retention time (3.85 min) and molecular ion (~1993 Da) close to the one of the LGTXs, the di-charged ion corresponding to feature 6 did not cluster with any toxin-deriving ions in the molecular network (purple node in Figure 4), and therefore could not be linked to a known compound.

Feature 8 clustered with features 3 and 4 in the molecular network (Cluster A, Figure 4), indicating shared fragments between these three mono-charged ions; however, their retention times were different, ranging between 3.92 and 4.99 min (Table 1). Therefore, the similarity in features 3, 4 and 8 is the result of structural similarities rather than different adducts of the same ion. It is reasonable to suggest that features 3 and 8 are by-products of feature 4 because it displays the highest molecular weight. Feature 4 shows many losses of water molecules during the fragmentation experiment (Table 1), and these losses may also be occurring prior to the analysis performed by UHPLC-HRMS. In this cluster A, feature 8 is linked to a fourth ion at *m*/*z* 717.4692, mainly found in the PHOT samples (yellow label) but not in the BACT samples, suggesting that it may be a catabolite of feature 8 resulting from photolysis.

The PLS-DA analysis showed that both LGTX-a and –c are among the most discriminant compounds in the treated extracts, suggesting a prevalent degradation of these toxins by bacteria compared to the other precursor toxins. This was not the case after exposure to sunlight as none of the known toxins were listed within the top variables of importance in the PLSDA (Table 1). None of the ions lost as a result of the PHOT treatment were identified and most were monocharged ions that were present as singletons in the molecular network (Table 1, Figure 4); only features 5 and 7 were present within a cluster.

The list of features that were gained after exposure to either bacteria or sunlight was much shorter with only two and four ions for the BACT and PHOT samples, respectively (Table 1). Most features were minor monocharged ions with no MS^2^ data acquired during the analysis which was performed in the data dependent acquisition mode. Feature 2 in the BACT samples eluted at 4.60 min and was the [M + Fe]^2+^ adduct of the molecular ion at *m*/*z* 1722.7990. Despite a successful fragmentation, neither this adduct nor the major [M + H + Na]^2+^ appeared in the molecular network, and thus it could not be linked to any known compound. Di-charged ions deriving from multiple losses of water molecules and a few monocharged ions (see diagnostic fragments in Table 1) constituted the main fragments.

Feature 1 in the PHOT samples was an [M + H]^+^ ion eluting at 9.26 min and therefore corresponds to a compound of mid-polarity. The molecular formula C_18_H_28_O_4_ was proposed by the SIRIUS software (100% score) and corresponds to an oxidized fatty acid type molecule. This ion clustered with others in the molecular network (Cluster C, Figure 4), but not with known toxins.

In cluster B, several monocharged ions corresponding to high molecular weight non-polar compounds (>1000 Da, RT > 10 min) grouped together. This family of compounds was mainly present in the BLK samples and could not be unequivocally identified in the absence of significant MS^2^ fragments. However, matches with phosphatidylinositol phosphate (PIP) and phosphatidyglcyerol (PG) with a delta ppm < 0.01 (LIPID MAPS ^®^ database) suggest they belong to a phosphorylated lipid group. Two more polar compounds (RT < 3.1 min), mainly present in the BACT samples, clustered with these lipids.

Other high molecular weight compounds (HMW, >2000 Da) eluting between 4.5 and 4.75 min are highlighted in the molecular network in the red rectangle (HMW rectangle, Figure 4), two of them being OVTX-a and OVTX-b were only detected in the Blk samples. One HMW feature, only detected in the PHOT and BACT samples, is a di-charged ion at *m*/*z* 1080.0504 eluting at 4.48 min. The corresponding molecular ion would present a Δ*m*/*z* = 16 between the LGTX-c ([M + 2H]^2+^ at *m*/*z* 1072.57), suggesting the presence of an additional oxygen atom. Three singletons corresponding to di-charged ions at *m*/*z* 797.49 and 769.41 were placed in the RVTX-type rectangle (Figure 4) given their close resemblance to the RVTX analogs.

## 3. Discussion

### 3.1. Toxin Content and Spontaneous Stability

Strains of *O.* cf. *ovata* isolated from the Ligurian Sea have been reported to produce a majority of OVTX-a, accounting for 56 to 76.8% of the total OVTX content, followed by OVTX-b (26%), while the other analogs (-c to –h) are present in minor amounts [13,50,51]. A high inter-strain variability is generally observed, with several analogs (OVTX-c, -f) not produced by some strains [51]. The Mediterranean MCCV54 strain used in this study has been shown to produce and release in the spent medium OVTX-a, OVTX-b and OVTX-d/-e [13] just like other Mediterranean strains [12]. However, in the present study, the toxin content of the spent medium was mainly composed by OVTX-a and -b and the three LGTX analogs (-a, -b, -c), while OVTX-d/-e were not detected. This might be because the experiment had been performed at the Scripps Institution of Oceanography where the strain was cultivated with aged Pacific seawater instead of Mediterranean seawater, which could impact and modify the intracellular metabolism and the subsequent extracellular release. The LGTX content cannot be compared to any other study since these analogs were only very recently described [8]. The stable concentration of the LGTX and OVTX over 62 h at room temperature in the glass vials used as blanks suggests that the toxins are not adsorbed on the glass walls. A preliminary experiment also ruled out toxin adsorption onto polypropylene (PP) surfaces (Figure S2). While toxin adsorption onto these surfaces cannot be completely excluded, these preliminary results indicate that it is insignificant compared to their potential transformation. The absence of transformation of the two toxins families in the absence of biotic or abiotic action is similar to the polyether ladder toxin PbTx 2 produced by the toxic dinoflagellate *Karenia brevis* [41] and domoic acid produced by the toxic diatom *Pseudo-nitzchia* spp. [43].

### 3.2. SDBL Cartridges Offer the Best Compromise for the Extraction of O. cf. ovata’s Toxins

Marine dissolved organic matter (DOM) is commonly extracted from seawater using styrene divinyl-benzene (DVB) PPL cartridges, following the procedure developed by Dittmar et al. [48]. The latter study recommends acidifying the samples (pH 2, HCl) to increase the DOM extraction efficiency. However, acidification of the samples did not improve the extraction efficiency of OVTX or LGTX, in line with recent findings on extraction of xenobiotics from seawater samples [52]. Furthermore, acidification significantly decreased the ability of most sorbents to extract OVTX-a and LGTX-a, -b and -c from the spent medium. OVTX analogs possess a terminal primary amine that becomes protonated under acidic conditions, therefore influencing extraction efficiency by these sorbents. Additionally, we used formic acid instead of HCl in these experiments because preliminary work revealed a complete degradation of OVTX in presence of HCl (Figure S3). This highlights the risk of using HCl acidification in DOM extraction for annotation purposes, especially when working with marine phycotoxins.

Our study clearly showed that the polymeric sorbents (HLB, StrataX, SDBL) gave better extraction efficiency for OVTX and LGTX analogs over C_18_-based ones. Previous work had divergent findings with comparable recoveries reported for OVTX-a using StrataX (35%) and BondElut C18 (40%) [49]. Among polymeric phases, the SDBL offered the best compromise between toxin extraction efficiency, extraction speed and reproducibility. The SDBL and the PPL sorbents have a comparable chemistry, both derived from a DVB polymer. Yet, the extraction yields obtained with PPL were significantly lower for all toxins except for LGTX-c, and likely results from the modifications applied to the DVB polymer in PPL sorbents. The extraction efficiency achieved with the two other polymeric sorbents HLB and StrataX was not significantly lower than for the SDBL sorbent; however, these two phases did not provide good reproducibility and had a longer processing time. Despite similar functional groups, the StrataX and the HLB sorbents did not display the same behavior; this difference might be attributed to the differences in their particle sizes (28–34 µm in StrataX against 50–65 µm in HLB).

### 3.3. Rapid Transformation of OVTX and LGTX by Bacteria

Both the LGTX and the OVTX half-lives were reached after 10 h of incubation, suggesting a rapid transformation by bacteria, possibly due to their high oxygen content [5,8]. Indeed, bacteria are known to preferentially target oxygen-rich molecules over several days [53,54], explaining their affinity for highly hydroxylated molecules like OVTX and LGTX [5,8] as confirmed by the untargeted approach. These transformation rates are comparable with those of saxitoxin and neosaxitoxin obtained after 12 to 24 h (>90%, [29]), but were much quicker than those obtained for gonyautoxin [29], PbTx-2 [30] or microcystin-LR [31]. Other studies on PbTx-2 suggest a rapid (<24 h) microbial breakdown that is mediated by microalgae and not bacteria [55,56]. However, the role of bacteria in producing lytic extracellular compounds was not investigated [56].

The transformation kinetics observed for the OVTXs was more rapid, suggesting that these molecules are more labile than the LGTXs. Even though the OVTXs were not listed among the main discriminant features by the untargeted approach, these palytoxin analogs might be hidden behind the features 3, 4 and 8 (cluster 1). Indeed, a side experiment that extracted an authentic palytoxin standard inoculated on a quartz filter with methanol in a sonicator bath revealed the emergence of an additional [M + H]^+^ ion at *m*/*z* 865.55 with a corresponding [M + 2H]^2+^ at *m*/*z* 433.24 (Figure S4). This emerging ion is therefore suspected to be a breakdown product of palytoxin, similar to the OVTXs. A perfect match of feature 8 in cluster 1 with this emerging ion suggests that the two other ions in this cluster could also be fragments of OVTXs. This hypothesis is supported by the large number of water losses during fragmentation of these three features, similar to the OVTX analogs [16,50].

Bacterially induced modifications to the molecular structures of marine dissolved organic matter are suspected to target functional groups and cause small changes in elemental composition and mass [54]. Small structural modifications of the OVTXs and the LGTXs should have appeared in the molecular network because molecules that are structurally related cluster together by this algorithm [57]. However, neither the OVTXs nor the LGTXs clustered with potential by-products, suggesting these two families, which are of high molecular weight and complex structure [5,8], may undergo more significant structural modifications that are not easily perceived by the molecular networking tool.

Transformed products from microalgal toxins have generally not been identified but were shown in some cases to form minor peaks with low intensities, such as for saxitoxin or gonyautoxin [29]. Similarly, the by-products of LGTXs and OVTXs that derived from bacterial metabolism must be minor compounds because only two significant features were highlighted by the untargeted analysis. One of the features gained during incubation was the doubly charged [M + Fe]^2+^ ion (*m*/*z* 888.8663) of the monoisotopic ion 1721.7993 Da. Interestingly, two fragments of this ion matched those in the features of cluster 1, indicating that they may be structurally related. However, the number of matching fragments was too small to form a cluster. If cluster 1 is a proxy for OVTX, the di-charged [M + Fe]^2+^ would therefore be an OVTX catabolite. The large number of water losses during fragmentation for this gained ion supports its structural resemblance to OVTX, which has numerous hydroxy groups.

A small number of studies have identified bacterial strains that are efficient in degrading microalgal phycotoxins. *Chromohalobacter* spp. were shown to degrade PbTx-2 [30]. Toxigenic bacteria are commonly related to alpha- and beta-proteobacteria, and most particularly members of the *Rhodobacteraceae* family [58]. Three alpha-proteobacteria form the most abundant genera of the bacterial consortium of *O*. cf. *ovata* during the exponential phase of growth [59]. In particular, two *Dinoroseobacter* and the *Roseovarius* species belonging to the *Rhodobacteraceae* family may have the potential to degrade the OVTXs and LGTXs. Similar bacterial communities have been reported during a natural bloom of *O*. cf. *ovata* in the Mediterranean Sea, with a strong presence of members of the *Rhodobacteraceae* [60], and thus would be expected to promote a similar bio-degradation of these toxins.

### 3.4. OVTXs Are more Sensitive to Light Than LGTX

A clear difference in photolytic instability was observed for analogs in the OVTX and LGTX families. Half-lives of the OVTXs were reached after 36 h of incubation, and most likely even earlier. The high sensitivity of the OVTX to light is similar to the rapid photolysis (ranging from 1 h to 96 h) observed for the cyanotoxin anatoxin-a (*Anabaena* sp. [40]), PbTx2 (*K. brevis* [41]) or domoic acid (*P. nitzchia* [42,43]). The LGTXs appeared less sensitive to light, although they do show some sign of degradation after 3 days of exposure to sunlight (~30%). Other cyanotoxins, such as cylindrospermopsin and nodularin a, have been shown to require a longer time for their photolysis with half-lives reached after about 10 days of exposure to artificial sunlight [61,62]. Degradation of microcystin-LR requires an even longer amount of time under natural sunlight conditions [63].

Chromophoric molecules like OVTX [5] absorb light in the UV and visible regions (170–740 nm) and thus are more prone to photochemical reactions [64]. The absence of a chromophore in the LGTX structures [8] is most likely responsible for their enhanced stability under natural sunlight.

The photolysis of several cyanotoxins has been shown to increase in the presence of reactive intermediates and through the process of photolysis sensitizers (anatoxin-a [65]; microcystin-LR [63]; nodularin [62]; cylindrospermopsin [66]). Dissolved organic matter (DOM) is the primary photosensitizer in seawater [67] and may include reactive oxygen species [68], reactive halogen species [69], carbonate radicals [38] or photosynthetic pigments [65]. In our experiment, the waterborne toxins originated from a DOM-enriched microalgal culture and were solubilized in aged natural seawater collected from the Pacific Ocean. Therefore, the experimental incubations contained both microalgal-induced and in situ DOM. Although OVTX are polyketides and not peptide assemblages like most cyanotoxins, the high DOM content with associated photolysis sensitizers may contribute to the degradation of these analogs.

Photodegradation of biotoxins can cause reversible rearrangement products for anatoxin-a [65] and domoic acid [70] as well as irreversible decarboxylation of domoic acid [70,71]. The formation of eighteen chemical entities was reported from the photodegradation of PbTx2 [41]. Despite the high degradation level observed for OVTX, no by-products were unambiguously identified by the untargeted metabolomics analysis or the molecular networking approach. None of the ions included in the short list of gained features from photolysis could be linked to OVTX. Most were minor monocharged non-polar ions (retention time >7.3 min) and none of their fragments matched with known OVTX fragments. Little is known about the oxidative reactions that may modify the OVTX or LGTX structures. The original structural elucidation of palytoxin (OVTX analog) was partly facilitated by a periodate oxidation reaction [72,73]. This oxidative reaction targets vicinal diols located near the terminus of the palytoxin molecule [72,74] or in its central portion [72]. The resulting cleavage of carbon–carbon bonds generates aldehyde-containing fragments [74] that are more polar and therefore have an earlier elution (ions at *m*/*z* 300.2 and 343.2 [74]). In the present study, no such ions were identified in the photolyzed samples; nevertheless, it is possible that photolysis produces other undescribed oxidized substances. The numerous hydroxy groups present in OVTX along with the two dienes and three olefins are expected to facilitate a variety of dehydration and oxidative reactions, leading to many potential transformed structures.

## 4. Material and Methods

### 4.1. Microalgal Culture

A monoclonal strain of *O*. cf. *ovata* obtained from the MCCV (Mediterranean Culture Collection of Villefranche, MCCV54) was grown in L1 medium [75] prepared with autoclaved aged and filtered natural seawater (collected from the Scripps Institution of Oceanography pier), adjusted to a salinity of 38. The cultures were maintained during ten days at 23 °C, under a 14/10 h light/dark cycle, with a light intensity of 250 µmol m^−2^ s^−1^ (Incubator Sanyo^TM^). Ten days of growth was previously identified as peak of diversity of the chemicals exuded by this dinoflagellate strain [14].

### 4.2. Experimental Set up

#### 4.2.1. Optimization of the Toxin Extraction

A volume of 15 L was grown for 10 days and the supernatant was retrieved by centrifuging the culture at 1800 rpm for 12 min. An additional gentle filtration of the supernatant through a 0.2 µm filter allowed removal of any living organisms. This spent medium was used to test the extraction efficiency of five types of sorbent, obtained from various manufacturers: two styrene divinyl benzene polymers (**PPL**—200 mg, 6 cc, Agilent, Santa Clara, CA, USA; **SDBL**—200 mg, 6 cc, Phenomenex, Torrance, CA, USA), an octadecyl bonded phase (**C18U**—200 mg, 6 cc, Phenomenex, Torrance, CA, USA) and two polymeric phases (**StrataX**—200 mg, 6 cc, Phenomenex; **HLB**—150 mg, 6 cc, Waters, Milford, MA, USA).

The 15 L were aliquoted in order to have 3 × 500 mL non-acidified and 2 × 500 mL acidified samples for each sorbent. Samples were acidified with a 0.1% vol/vol addition of formic acid (FA, LiChropur^®^, LC-MS grade, Millipore, Burlington, MA, USA). The cartridges were previously activated with MeOH and equilibrated with MilliQ water. After sample loading, each cartridge was washed with two column volumes of MilliQ water, and the extract was eluted with two column volumes of MeOH (LC-MS grade, Alfa Aesar^®^, Tweksbury, MA, USA). For pH-modified samples, the MilliQ water (equilibration and washing steps) was adjusted to pH 2 using formic acid and the elution was also performed with MeOH. The MeOH eluates were evaporated down to a volume of 150 µL with a rotavapor followed by a gentle nitrogen evaporation, and then stored at −20 °C until analysis by UHPLC-HRMS. Before analysis, the eluates were centrifuged at 4000 rpm for 5 min at 4 °C to remove any crystallized salts that formed under the combined effect of cold and storage in MeOH.

#### 4.2.2. Biotic and Abiotic Transformation of the Toxins

In order to mimic the natural occurrence of waterborne OVTX, LGTX and RVTX during *O.* cf. *ovata* blooms, spent culture medium was used to assess the transformation of the phycotoxins. A 22 L culture was grown over 10 days and the supernatant was retrieved by centrifuging the cultures at 1800 rpm for 12 min. The supernatant was further filtered gently through 0.2 µm to remove micro-organisms and particles/debris. This spent medium was extracted without pH adjustment using a SDBL cartridge (200 mg, 6 cc, Phenomenex), based on the results obtained during the optimization step. Each cartridge was used to extract a volume of 1.5 L (*n* = 15 cartridges) following the procedure described above. All eluates were combined and evaporated using a rotavapor until only water remained in the flask. This aqueous extract was further lyophilized to avoid toxin losses that might occur during evaporation. The extract was then resuspended in 22 mL of L1 medium. A 9 L L1 media blank was processed following the same procedure and resuspended in 9 mL of L1 medium.

A volume of 700 µL of the toxins extract was used to inoculate the different treatments: (i) Toxin blanks (BLK), (ii) Photolysis (PHOT) and iii) Bacteria (BACT). All treatments were performed in triplicate, for a total of 9 flasks per treatment. Sampling was performed after an incubation of 10 h (T1), 36 h (T2) and 62 h (T3). Each replicate was sampled for all three time points for all treatment. This sampling scheme allowed an exposure to sunlight for three consecutive days for the PHOT samples. Unfortunately, the PHOT samples at 10 h were lost.

Toxin blanks (BLK) consisted of 20 mL of sterile L1 medium in glass vials incubated in the dark at room temperature (24 °C). The bacterial treatment (BACT) consisted of 50 mL of sterile L1 medium in 250 mL PC flasks previously acid-washed to which bacteria were added. The bacterial community used for this treatment was obtained from a culture of *O.* cf. *ovata* at 14 days (stationary phase); three 100 mL flasks were filtered through three 3 µm filters to remove microalgal cells. The filtrates were further filtered on nine 0.2 µm filters in order to collect free living bacteria. The 0.2 µm filters were soaked in the nine PC flasks used for seeding the experiment. The PC flasks were kept at 24 °C in the incubator under the same light/dark cycle used for the microalgal culture. The photolysis treatment (PHOT) consisted of 20 mL of sterile L1 medium in quartz beakers maintained outside in a running seawater tank to allow full solar UV radiation at a fairly constant temperature (20–24 °C). At the time and the coordinates of the experiment (18–20 August 2020; 32°51′56″ N, 117°15′13″ W), the photosynthetically active radiation (PAR) varied between 107.96 and 119.5 W/m^2^, and the natural light cycle was 13/11 h. For comparison, in the North-Western Mediterranean Sea where recurrent blooms of *Ostreopsis* occur, the PAR is often comprised between 120 and 160 W/m^2^ over the duration of the bloom.

### 4.3. Sample Treatment

At each sampling time, three vials or flasks were collected and the sample volume was extracted with a single-use SDBL cartridge, following the procedure previously described above. The eluates were dried down to 150 µL with a gentle stream of N_2_ and were stored at −20 °C. A preliminary filtration on a 0.2 µm filter was performed for the BACT samples to remove all bacteria from the medium. Additionally, a volume of 1.5 mL of the BACT treatment was collected at each sampling time, fixed with 30 µL of formaldehyde for bacterial abundance (2% vol/vol) and stored at −80 °C until analysis.

### 4.4. Chemical Analysis

Before analysis, the samples were centrifuged at 4000 rpm for 5 min to pellet down any crystalline salts. The chemical content of the different samples was analyzed by Ultra High-Performance Liquid Chromatography coupled to High Resolution Mass Spectrometry (UHPLC-HRMS) using a Thermo Scientific Vanquish system (Agilent Technologies, Santa Clara, CA, USA). Separation was achieved on a UPLC C18 column (Kinetex^®^ 2.6 µm, Polar C18, 150 × 2.1 mm, Phenomenex) maintained at 40 °C. Eluent A was milli-Q water and Eluent B was acetonitrile (ACN, LC-MS grade, Alfa Aesar^®^), both eluents containing 30 mM of anhydrous acetic acid. A gradient elution of 20 to 99% B over 10 min was set to allow the detection of both the toxins and their potential degradation products. The flow rate was 500 µL min^−1^ and the injection volume was 10 µL. Mass spectrometry data were acquired using an Orbitrap Elite MS mass spectrometer (Agilent Technologies, Santa Clara, CA, USA) equipped with an electrospray ionization in the positive mode. Full scan spectra were acquired in the range 200–2000 Da, in the CID mode with a Fourier Transform Mass Spectrometry (FTMS) Analyzer set at a resolution of 120,000. The MS/MS spectra were acquired in the dependent scan mode using collision energies (CE) of 35 eV. The capillary voltage of the MS spectrometer was set at 3500 V (positive mode), and the nebulizing parameters were set as follows: nebulizing gas (N_2_) pressure at 0.5 bar, drying gas (N_2_) flow at 11 L min^−1^, the drying temperature at 300 °C and the Vaporizer/Sheath Gas Temp, 300 °C.

In the absence of authentic standards to quantify the toxins concentrations, the comparison between the samples was based on the relative intensity of the most representative ion of a toxin (e.g., the most intense). The following ions were used: [M + 2H − H_2_O]^2+^ = 1315.2489 (OVTX-a), [M + 2H − H_2_O]^2+^ = 1337.2633 (OVTX-b), [M + 2H]^2+^ = 987.0349 (LGTX-a), [M + 2H]^2+^ = 1043.0673 (LGTX-b) and [M + 2H]^2+^ = 1072.0673 (LGTX-c).

To perform untargeted metabolomics on all treatments, a random injection of the samples on the UHPLC-HRMS was performed to avoid systematic errors. Four quality control samples (QCs) were prepared by mixing 2 µL of each sample and were injected every seven samples. Analytical blanks were also prepared and injected at the beginning and the end of the sequence. Raw UHPLC-HRMS data were analyzed using Xcalibur^®^, converted into mzXML files using MSconvert [76], and processed for mass detection, building chromatograms, deconvolution, isotope finding and alignment, using the open source MZmine (version 2.53, [77]). Subsequently, the features were submitted to the GNPS platform [57] to perform a feature-based molecular networking (FBMN, [78]) that improves clustering of compounds of similar chemical structure in order to enhance the detection of potential toxin by-products.

### 4.5. Bacterial Abundance

The bacterial abundance was measured by flow cytometry. Bacterial cells were stained with SYBR Green I at 0.025% (vol/vol) final concentration [79], and counts were performed on a Accuri C6Plus flow cytometer (Becton Dickinson©, Franklin Lakes, NJ, USA).

### 4.6. Statistical Analysis

To evaluate the statistical differences between groups, the non-parametric *t*-test was chosen and run using Rstudio^®^.

## 5. Conclusions

While the OVTXs and LGTXs are stable compounds under protected conditions (dark, sterile), they are fragile molecules prone to natural biotic and abiotic transformation. In particular, the half-lives of the OVTX analogs are very short under sunlight conditions or in presence of *O*. cf. *ovata*’s bacterial consortium in culture (<10 h). The different levels of LGTXs and OVTXs tranformation observed under both biotic and abiotic conditions suggest that different transformation pathways might be at play, and therefore confirm that they likely belong to distinct chemical families [8].

This result raises important questions on the presence of waterborne OVTXs, their transfer to sea spray aerosols and their toxicity to coastal populations. Several outbreaks along the Mediterranean and more recently along the French Atlantic coastlines [80] have been attributed to the aerosolization of OVTX analogs, although these toxins have rarely been detected in sea spray aerosols. The short half-lives of these phycotoxins under biotic and abiotic conditions implies a reduced longevity as waterborne biomolecules, reducing their transfer to the sea spray aerosols. In circumstances where the OVTXs are aerosolized, they would necessarily be exposed to UV irradiation and radical species, and would therefore be rapidly degraded. As a result, the present results challenge the concept of aerosolization of the OVTXs as a cause of toxic inhalations in natural ecosystems. However, if not these toxins, which compounds are responsible for the reported dermatitis and toxic inhalation? The assessment of the allergic potential of the transformed products of OVTX and LGTX represent one possible area for future investigation; however, the identities of these would first need to be thoroughly identified. This current study provides preliminary indications that the dicharged [M + Fe]^2+^ ion at *m*/*z* 888.8663 is a potential by-product of the OVTX analogs. All other catabolites are most likely smaller compounds devoid of major structural similarities with the OVTXs and LGTXs. This necessarily precludes the establishment of a link between these compounds and potential toxin precursors, even using cutting-edge tools like GNPS molecular networking.

## Figures and Tables

**Table 1:** Most significant features obtained from the PLS-DA, driving the distribution of the BACT and PHOT samples compared to the BLK samples. The features are organized as lost or gained features in the table. Molecular formulae were calculated using SIRIUS^®^ and are given with a score (%). The inclusion of each feature in the molecular network is indicated in the GNPS (Global Natural Products Social molecular networking) column. Diagnostic fragments are given for each feature. Known toxins are indicated in the molecular formula column.

		Feature	m/z	RT	Ion	Molecular Formula	Score Formula (%)	GNPS	Diagnostic Fragments
**BACTERIA**	Lost	1	1072.0619	4.48	[M + 2H]^2+^	LGTX-c	-	singleton	1057.55 ( + losses water); 979.50 ( + losses water); 655.31; 590.78; 350.47
		2	647.4052	5.79	[M + H]^+^	C_39_H_54_N_2_O_6_	85.79	singleton	629.39; 603.41; 550.82; 497.23; 456.27; 398.26; 316.22; 250.14; 192.13
		3	997.6001	4.59	[M + H]^+^	nd	-	cluster	920.46; 911.45; 902.45; 893.44; 884.44 (all dicharged = loss water); 865.55; 464.26; 420.23; 360.21; 302.21
		4	1013.5953	3.92	[M + H]^+^	nd	-	cluster	881.55; 464.26; 420.23; 360.21; 302.21
		5	459.3292	13.74	[M + Na]^+^	C_23_H_48_O_7_	85.3	cluster	415.29; 403.20; 371.27; 345.24; 305.15; 275.20; 261.11; 217.09
		6	997.5004	3.85	[M + 2H]^2+^	nd	-	singleton	1618.81?; 1098.55; 979.98; 824.39; 842.39; 806.37; 466.22; 448.21; 430.20; 360.21; 306.17
		7	1007.5271	4.64	[M + 2H]^2+^	LGTX-a	-	-	989.51 ( + water losses); 920.46 ( + water losses); 652.26; 540.61; 351.35
		8	865.5573	4.99	[M + H]^+^	C_45_H_76_N_4_O_12_	52.02	cluster	847.54; 643.43; 602.40; 560.35; 464.26; 420.23; 360.21; 302.21; 244.17
	Gained	1	863.4252	8.85	[M + H]^+^	nd	-	-	
		2	888.8663	4.60	[M + Fe]^2+^	nd	-	-	823.9066; 814.8993; 805.89; 796.89; 787.88; 778.89; 670.8027; 521.73; 464.26; 420.23
**PHOTOLYSIS**	Lost	1	343.0887	2.36	[M + Na]^+^	C_11_H_20_N_4_O_3_S_2_	59.46	singleton	336.06; 319.01; 277.00; 258.99; 240.98
		2	997.5004	3.85	[M + 2H]^2+^	nd	-	singleton	1618.81?; 1098.55; 979.98; 824.39; 842.39; 806.37; 466.22; 448.21; 360.21;
		3	329.1102	0.76	[M + Na]^+^	C_13_H_24_NO_3_S_2_	80.35	singleton	299.09; 281.10; 253.11; 219.12; 167.09; 139.09; 122.07; 110.07
		4	647.4052	5.79	[M + H]^+^	C_39_H_54_N_2_O_6_	85.79	singleton	603.41; 550.82; 497.23; 456.27; 398.26; 316.22; 273.10; 192.13
		5	387.0881	2.36	[M + H]^+^	nd	-	cluster	no MS^2^
		6	865.5573	4.94	[M + H]^+^	C_45_H_76_N_4_O_12_	52.02	cluster	847.54; 643.43; 602.40; 560.35; 464.26; 420.23; 360.21; 302.21; 244.17
		7	285.2904	6.09	[M + H]^+^	C_17_H_36_N_2_O	100	cluster	263.0188; 240.2317
		8	271.2422	8.45	[M + H]^+^	C_20_H_30_	100	singleton	odd isotopic pattern
	Gained	1	309.2062	9.26	[M + H]^+^	C_18_H_28_O_4_	100	cluster	281.21; 277.17; 253.14; 221.111; 193.12; 175.11; 165.05; 115.03; 95.08; 85.02
		2	409.2070	8.26	[M + H]^+^	-	-		
		3	409.2070	9.26	[M + H]^+^	-	-		
		4	330.2275	7.29	[M + H]^+^	-	-		

## Data Availability

All UHPLC-HRMS data are archived and available at the following repository: www.ebi.ac.uk/metabolights/MTBLS6538.

## Supplementary Materials

The following supporting information can be downloaded at: https://www.mdpi.com/article/10.3390/md20120748/s1. Figures S1–S4: Additional information on the *O*. cf. *ovata*’s toxins behavior and lability
