# Supplementary material for: Rapid Biotic and Abiotic Transformation of Toxins produced by Ostreopsis. cf. ovata
Source: Mar Drugs. 2022 Nov 28;20(12):748. doi: 10.3390/md20120748 (PMC9787646; doi:10.3390/md20120748)
Supplement: Supplementary file 1 [file marinedrugs-20-00748-s001.zip › marinedrugs-1969580-supplementary.pdf]

Supplemental Information

Rapid Biotic and Abiotic Transformation of Toxins produced by *Ostreopsis.* cf. *ovata*

Eva Ternon [1,2,*], Olivier P. Thomas [3], Rodolphe Lemée [2] and William H. Gerwick [1,4]

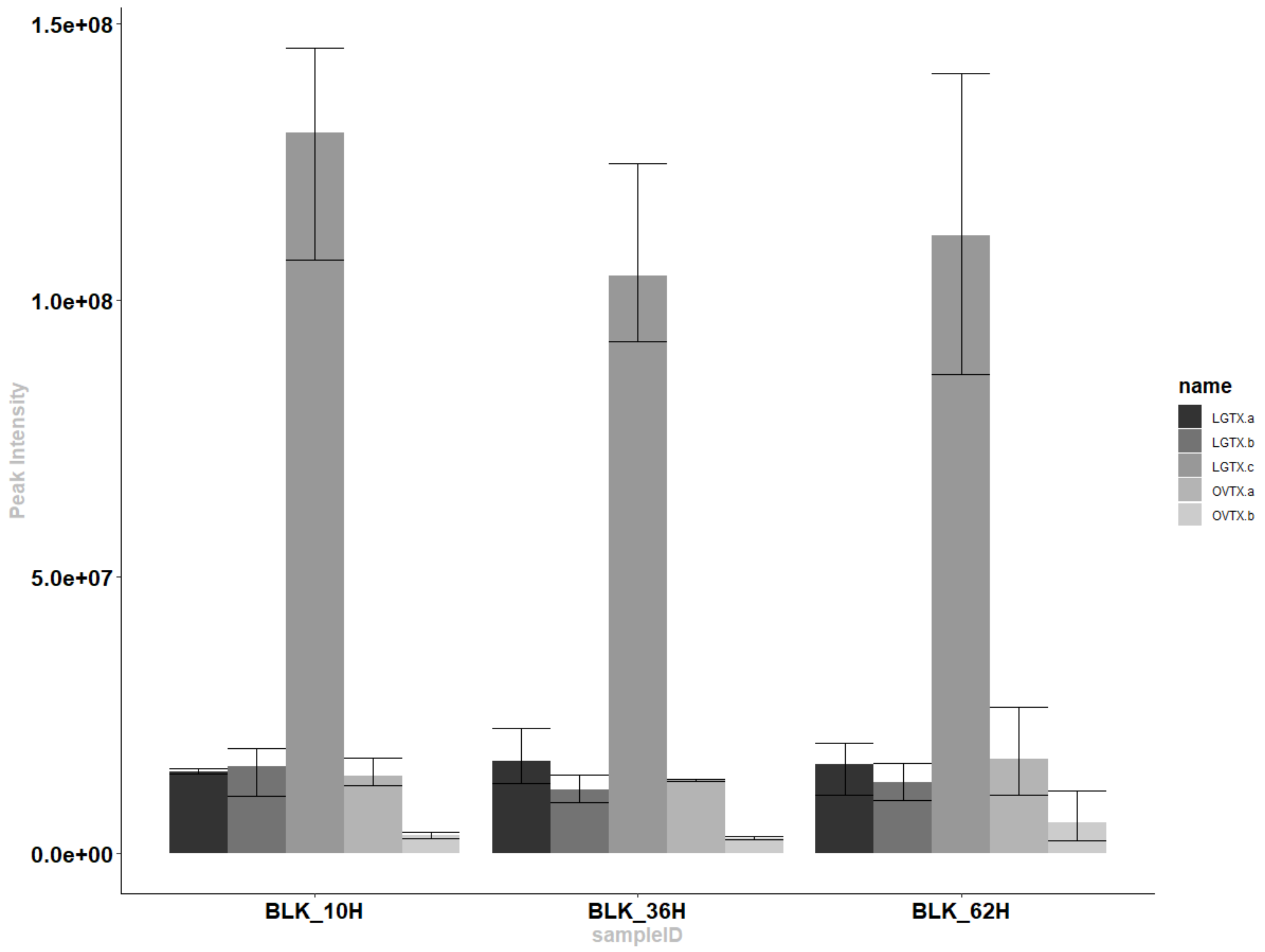

Figure S1. Peak intensity of OVTX and LGTX across time in the blank samples (BLK). Samples were kept in the dark at room temperature for 62 hours. The error bars were built with n=3 replicates.

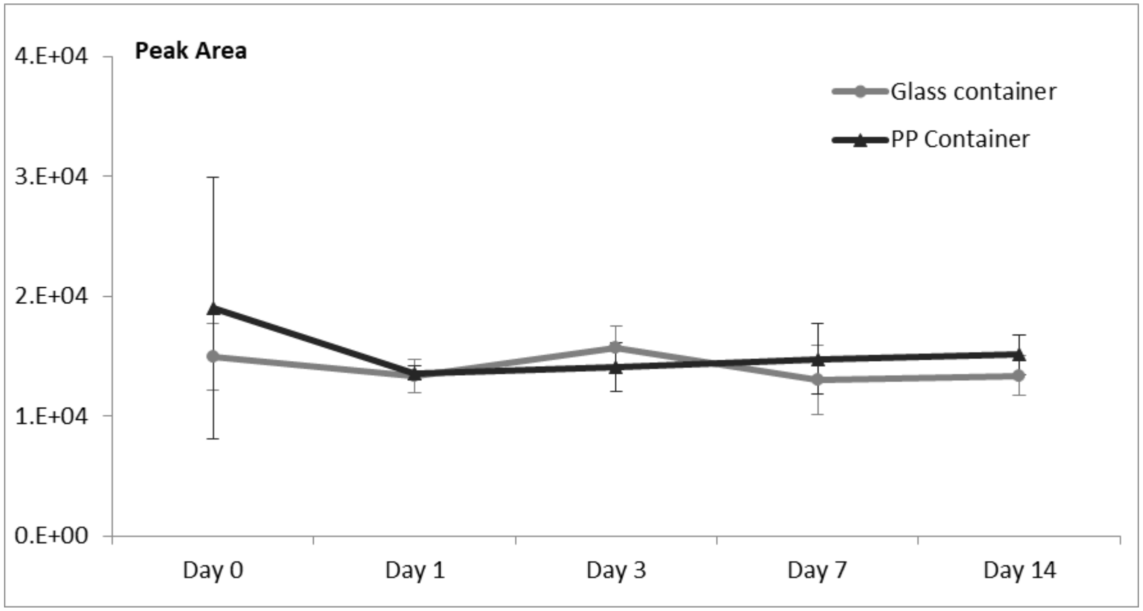

Figure S2. Peak area of the ion $[M+H+2Na]^{3+}$ at *m/z* 908.8091 of an authentic PLTX standard solubilized in MeOH and maintained at 4°C during 14 days in a glass (grey line) or polypropylene (PP) container (dark line). Replicate of each treatments (n=3) were prepared and sacrificied at each sampling time.

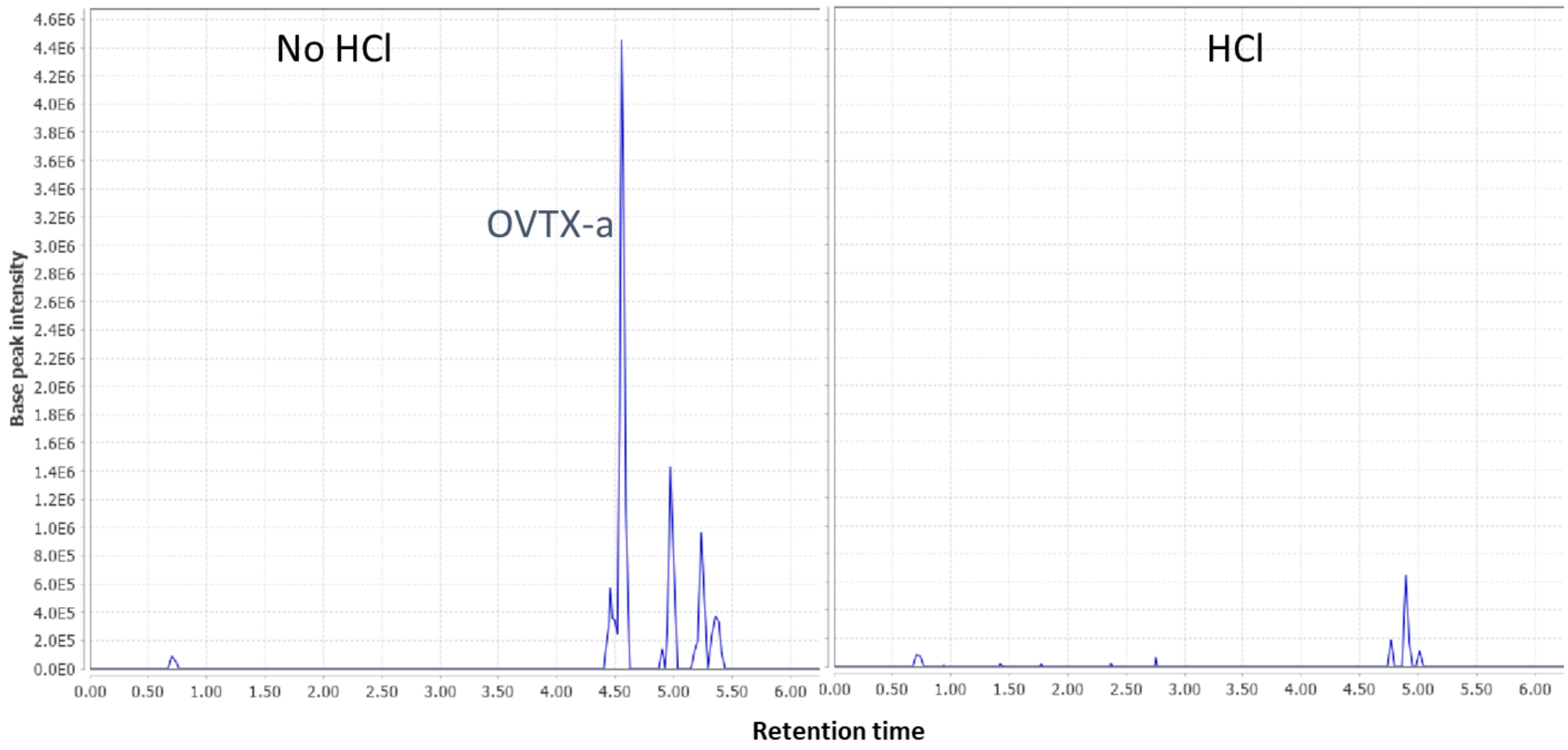

Figure S3. UHPLC-HRMS extracted chromatogram (*m/z* 1315.2546, OVTX-a) of the culture spent medium (left panel) without hydrochloric acid (HCl) addition, and (right panel) after addition of 0.1% of HCl following Dittmar et al., (2008) recommendations.

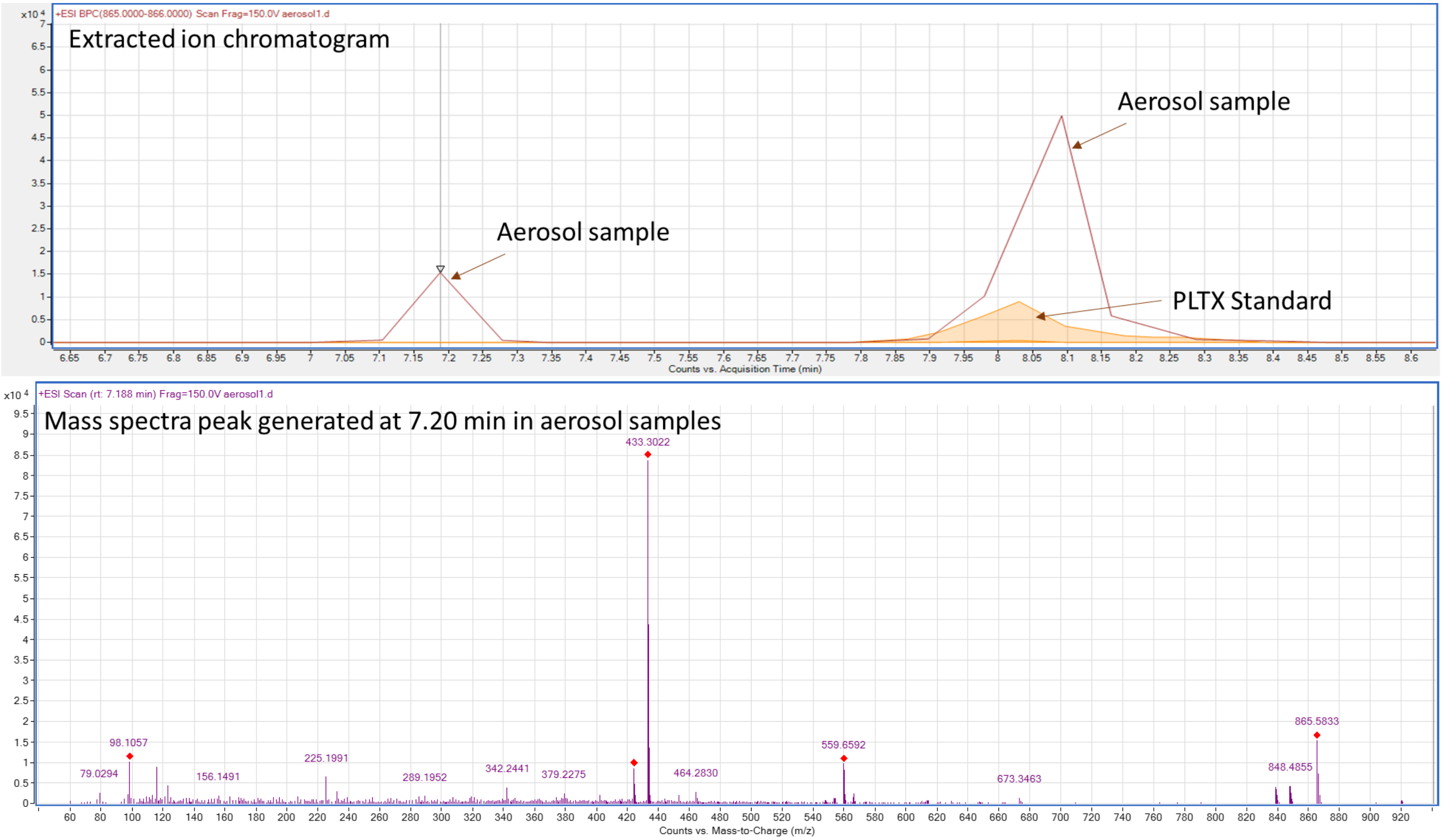

Figure S4. Extracted chromatogramm ion *m/z* 865-866 for an authentic PLTX standard (orange) and the same PLTX standard deposited on a quartz filter commonly used for aerosol sampling and further extracted in MeOH in a sonication bath for 5 min. The mass spectra corresponding to the new peak obtained after extraction is shown in the lower panel displaying one monocharged ion at *m/z* 865.5833 and its dicharged ion *m/z* 433.3022.
